# Quantitative measures of gingival recession and the influence of gender, race, and attrition

**DOI:** 10.1186/s40510-017-0199-4

**Published:** 2018-01-29

**Authors:** Chester S. Handelman, Anthony P. Eltink, Ellen BeGole

**Affiliations:** Department of Orthodontics, College of Dentistry, University of Illinois at Chicago, Chicago, IL USA

**Keywords:** Gingival recession, Gingival recession measures, Attrition, Gingival architecture, Cementum exposure, Clinical crown height

## Abstract

**Background:**

Gingival recession in dentitions with otherwise healthy periodontium is a common occurrence in adults. Recession is clinically measured using a periodontal probe to the nearest millimeter. The aim of this study is to establish quantitative measures of recession, the clinical crown height, and a new measure the *gingival margin-papillae measurement*. The latter is seen as the shortest apico-coronal distance measured from the depth of the gingival margin to a line connecting the tips of the two adjacent papillae.

**Methods:**

Measurements on all teeth up to and including the first molar were performed on pretreatment study models of 120 adult Caucasian and African-American subjects divided into four groups of 30 by gender and race.

**Results:**

Both the clinical crown height and the gingival margin-papillae measurements gave a *true positive* result for changes associated with gingival recession. Tooth wear shortens the clinical crown, and therefore, the measure of clinical crown height can give a *false negative* result when gingival recession is present. However, the gingival margin-papillae measurement was not affected by tooth wear and gave a true positive result for gingival recession. Tooth wear (attrition) was not associated with an increase in gingival recession. These measures are also useful in detecting recession prior to cemental exposure. Measures for recession and tooth wear were different for the four demographic groups studied.

**Conclusions:**

These measures can be used as quantitative standards in both clinical dentistry, research, and epidemiological studies.

## Background

Gingival recession is observed in adults otherwise free of periodontal disease with a high standard of oral hygiene [1]. Recession is of concern to patients who view the loss of attachment as a health issue as well as an esthetic problem. The intent of this paper is to establish quantitative measures of the gingival margin position in four groups: Caucasian and African-American males and females that were otherwise free of periodontal disease. The measures evaluated are clinical recession, clinical crown height, and a new measure the *gingival margin-papillae measurement*. The influence of dental attrition on these measures was also evaluated.

While researchers in periodontics have looked to understand the biology of the periodontium [2, 3], the fields of esthetic dentistry and orthodontics have been concerned with the role that gingival architecture plays in dental esthetics [4, 5].

In health, the shape of the papilla in a given interdental space depends on the contact point between the two adjoining teeth [6]. In the absence of interproximal bone loss, the gingival papilla completely fills the gingival embrasure [2]. As stated by Löe et al. [7] and Newman et al. [8], the gingival margin’s position in the healthy periodontium is 0.5 to 2.0 mm coronal to the cemento-enamel junction. As the cemento-enamel junction of the tooth curves apically from its more coronal interdental position, the gingival margin follows this curvature, creating the characteristic scalloping contour of healthy gingiva.

Age-related gingival recession observed in some subjects results in changes in the gingival architecture that can be distinguished from changes resulting from periodontal disease [1, 9, 10]. In cases of gingival recession that occurs in the absence of gingivitis or periodontitis, the papillae maintain its normal position while the curvature of the gingival margin on the facial aspect of the tooth deepens [9, 10].

Gingival recession is measured clinically as the distance from the cemento-enamel junction to the depth of the free gingival margin using the millimeter markings on the periodontal probe and reflects exposure of the root cementum [7, 8]. While this clinical recession measurement is useful in following the progression of gingival recession, it has two shortcomings. First, it is semi-quantitative with precision to 0.5 to 1.0 mm. Second, it is not an accurate assessment of the apical migration of the gingival margin due to its reliance on the cemento-enamel junction as a reference point. Given that the gingival margin’s physiological position may be 0.5 to 2.0 mm coronal to the cemento-enamel junction [7, 8], the reference point for clinical recession is obscured by the gingiva itself. Thus, the early stages of gingival recession prior to exposure of the cementum—defined in this paper as *prodromal recession*—might go undiagnosed.

Clinical crown height is a measure of the position of the gingival margin that could be used in determining the position of the gingival margin [11]. Powell and McEniery [12] showed that the norms for clinical crown heights are useful in the diagnosis of gingival recession.

The use of clinical crown height as a measure of gingival recession is complicated by the wear at the incisal edge or cusp tip. Attrition over time leads to a decrease in the clinical crown heights of patient’s teeth and can mask the presence of gingival recession if clinical crown height is used as a measure. One may also question if tooth wear which is usually attributed to bruxism plays a role in gingival recession possibly due to abfraction.

Given the complexity of assessing gingival recession, it can be proposed that a new measure of gingival recession should be established which would allow precise quantification of recession, one of the shortcomings of clinical recession. Gorman [13], Albandar and Kingman [14], and Löe et al. [15] found that interproximal recession in the absence of periodontal disease is rare and gingival recession occurs almost exclusively on the buccal surfaces of teeth at the depth of the gingival crest [7–9]. Thus, the shortest apico-coronal distance measured from the depth of the gingival margin to a line connecting the tips of two adjacent papillae—the gingival margin-papillae measurement—would serve as an accurate assessment of gingival recession. Prodromal recession—recession prior to cemental exposure would be detected, and enamel attrition would not confound the measurements.

## Methods

This retrospective study analyzed pretreatment study models of adult patients from a private orthodontic practice. In addition to study models, recording of probing depths, full mouth dental x-rays, and intraoral photographs were available on each subject. All records were de-identified per the HIPAA regulations to ensure that the patients could not be identified. This study was performed under the guidance of the *University of Illinois at Chicago's Institutional Review Board, approval (#2004-0437)*.

### Selection criteria

The objective of the selection criteria was to establish a group of subjects for the study of gingival recession that occurs in persons with otherwise periodontally healthy mouths. The study sample consisted of 120 patients chosen serially so that the requisite number of 30 subjects was achieved in each of the four demographic groups: Caucasian and African-American males and females.

The inclusion criteria were as follows:

1. Age of 20–59
2. No more than three areas of bone loss (3 mm or more from the interproximal cemento-enamel junction to the crest of the alveolus) evident on full mouth radiographs and no more than three teeth that had a pocket depth reading of 4 mm or more
3. No evidence of gingivitis as judged on intraoral photographs
4. Individual teeth with radiographic evidence of localized bone loss of 3 mm or more were excluded
5. Individual teeth with a pocket depth of 4 mm or more were excluded
6. Teeth blocked out of the arch or adjacent edentulous spaces were excluded
7. Fixed prosthodontic crown and fixed bridge abutments were excluded
8. Teeth subject to frenum pull to the gingiva were excluded

The number of subjects excluded because of criteria 1 to 3 was not recorded. Exclusion because of periodontal bone loss or deep probing measures numbered about 25 subjects. Only a few subjects were excluded because of gingivitis.

The following data for each subject were recorded: age in years, race, and gender. In order to evaluate subjects’ gingival health and cemental exposure, 35-mm film slides of each subject’s buccal and anterior teeth were mounted in a table viewer with × 5 magnification. Using the intraoral photographs as an aid in identification of the cemento-enamel junction , the following measurements were made for each tooth on study models: clinical recession, clinical crown height, gingival margin-papillae, and attrition.

The data for clinical crown height and gingival margin-papillae were measured and recorded using a Mitutoyo Absolute Digimatic digital caliper (No. 99MAD014M, Series No. 500; Mitutoyo American Corporation, Aurora, IL). An input tool with a connecting cable (No. 99MAM014B1, Series No. 264 and No. 959149; Mitutoyo American Corporation, Aurora, IL) was used for the automatic transfer of the data into the Microsoft Excel spreadsheet and transferred to the Statistical Package for the Social Sciences (SPSS, version 11.5; SPSS Inc., Chicago, IL).

### Measurements

**Fig. 1:**
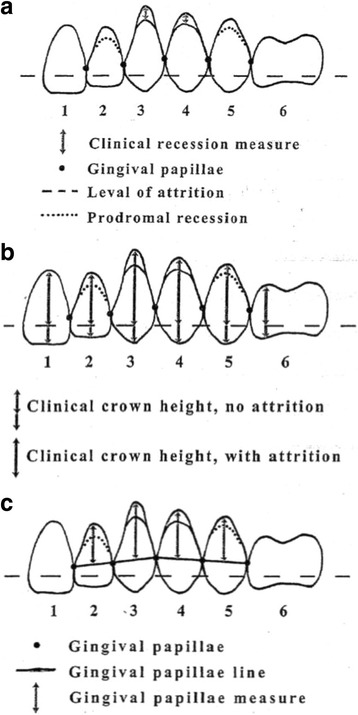
**a** Gingival recession is measured clinically as the distance in millimeters from the CEJ to the free gingival margin using a periodontal probe. **b** Clinical crown height is a measure from the cusp tip or incisal edge to the depth of the gingival sulcus. Attrition (long dash of horizontal line) will shorten this measure. **c** Gingival margin-papillae measure is the shortest apico-coronal distance measured from the depth of the gingival margin to a line connecting the tips of the two adjacent papillae. (Reprinted with permission from Eltink et al. [27])

Clinical recession was recorded on all teeth as the shortest distance from the cemento-enamel junction to the deepest curvature of the gingival margin (Fig. 1a). The measurements were made to the nearest whole millimeter using a standard periodontal probe, always rounding up so that even minimal cemental exposure was scored at 1 mm.

Clinical crown height was measured with digital calipers to the nearest one hundredth of a millimeter on the facial surface of each crown from the cusp tip or incisal edge to the deepest curvature of the gingival margin along the long axis of the tooth for bicuspid, cuspid, and incisor teeth and on the molar teeth at the mesiobuccal cusp (Fig. 1b).

The gingival margin-papillae measurements were recorded using the digital calipers to the nearest one hundredth of a millimeter as the shortest distance from the interpapillae line to the deepest curvature of the gingival margin (Fig. 1c). The measurements on molar teeth were made at the mesiobuccal cusp.

**Fig. 2:**
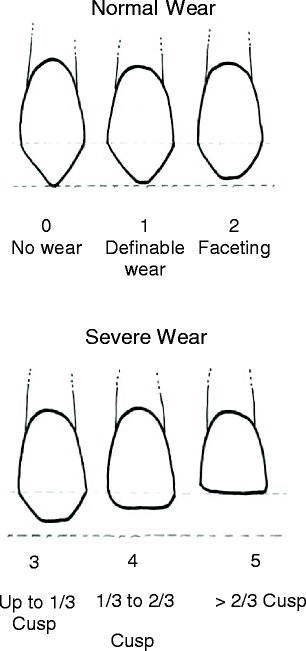
The Hooper index [16] is used to define normal wear (0–2) and severe wear (3–5) teeth. The dashed line indicates the crown height prior to tooth wear

Tooth wear was scored on the buccal of all teeth using Hooper et al.’s tooth wear index [16]. Each tooth was assigned a score from 0 to 5 according to the criteria outlined by Hooper et al. (Fig. 2).

### Statistics

A power analysis using data from a previously published thesis [17] indicated that with a sample of at least 30 subjects in each group, the study has a power of at least 80%, (type error of 5%) to detect 1 standard deviation mean difference between the groups.

Means and standard deviations of all measures were established for each tooth class from the central incisors to and including the first molars. Independent *t*-tests show no significant difference between values for the right and left sides for each tooth class so these values were combined. Independent *t*-tests were used to evaluate if differences existed between the subsets: the recession and non-recession teeth and the tooth wear and non-tooth wear teeth.

The age data for the study sample were analyzed to determine if the four demographic groups differed with respect to age. The results of the ANOVA comparison revealed that there was no difference in age among the groups using *p* < 0.05. The mean age for the four groups ranged from 33.2 to 39.2 years [17].

The accuracy of the four measurements was evaluated in ten randomly selected cases. Study models were measured and then re-measured 10 days later. Using the Pearson correlations, all re-measured sets of values showed a significant correlation at the 0.01 level. All measurements and statistics were performed by one investigator (APE).

## Results

Mean values and standard deviations for the measurement of clinical crown heights for each tooth are listed for the four demographic groups in Table 1. The mean values and standard deviations for the measurement of gingival margin-papillae are listed in Table 2. The overall data set of 120 subjects (*N* = 1522 teeth) was separated into two subsets on the basis of the clinical recession measurement for each tooth. The first subset, called the “non-recession teeth” (*N* = 1222 teeth), was comprised of all teeth whose measurements were scored 0 mm for the clinical recession measurement. The second subset, called the “recession teeth” (*N* = 300 teeth), was comprised of measurements on all teeth that were scored 1 mm or greater including teeth with minimal recession. Independent *t*-tests were performed for clinical crown height and gingival margin-papillae to determine whether differences exist between these two subsets of teeth for these measurements.

**Table 1:** Clinical crown height means ± SD for the subgroups

Tooth class	Caucasian				African-American				Total (*N* = 120)	
	Male (*N* = 30)		Female (*N* = 30)		Male (*N* = 30)		Female (*N* = 30)			
	Mean (mm)	SD	Mean (mm)	SD	Mean (mm)	SD	Mean (mm)	SD	Mean (mm)	SD
U6	7.20	1.04	6.68	0.93	7.08	1.05	6.07	1.04	6.74	1.10
U5	7.60	0.93	7.21	1.07	7.18	0.87	6.64	0.82	7.16	0.98
U4	8.46	1.03	8.20	0.90	8.27	0.86	7.72	0.83	8.16	0.94
U3	10.49	1.15	9.80	1.00	10.22	1.05	9.50	1.02	10.00	1.11
U2	8.97	1.05	8.96	1.06	8.79	0.98	8.50	1.06	8.81	1.04
U1	10.49	1.22	9.96	0.81	10.60	0.98	10.02	1.23	10.27	1.10
L6	7.08	0.64	7.03	1.22	6.49	0.63	6.30	0.79	6.73	0.89
L5	7.54	1.01	7.54	0.89	7.41	0.90	7.09	0.78	7.40	0.91
L4	8.81	0.95	8.34	0.59	8.48	0.74	7.68	0.64	8.33	0.85
L3	10.40	1.15	9.71	1.14	10.00	1.20	9.03	1.25	9.78	1.27
L2	8.29	0.99	8.57	0.99	8.27	0.87	8.14	1.23	8.32	1.03
L1	8.15	1.17	8.33	0.99	8.24	0.91	8.13	1.33	8.21	1.10

Reprinted with permission from Eltink et al. [17, 27]

**Table 2:** Gingival margin-papillae means ± SD for the subgroups

Tooth class	Caucasian				African-American				Total (*N* = 120)	
	Male (*N* = 30)		Female (*N* = 30)		Male (*N* = 30)		Female (*N* = 30)			
	Mean (mm)	SD	Mean (mm)	SD	Mean (mm)	SD	Mean (mm)	SD	Mean (mm)	SD
U6	3.07	0.95	2.56	0.79	2.59	0.72	1.99	0.54	2.54	0.84
U5	3.03	0.83	2.77	0.91	2.47	0.56	2.12	0.51	2.60	0.79
U4	3.51	1.00	3.25	0.83	3.24	0.66	2.83	0.60	3.21	0.81
U3	4.86	1.24	4.47	0.75	4.38	1.08	3.77	0.83	4.37	1.06
U2	3.85	1.40	3.64	0.79	3.11	0.69	2.94	0.77	3.39	1.01
U1	4.37	1.71	3.84	0.67	3.80	0.61	3.54	0.69	3.90	1.07
L6	2.93	0.75	3.14	1.22	2.35	0.59	2.25	0.34	2.67	0.85
L5	3.00	0.73	3.22	0.85	2.75	0.62	2.49	0.53	2.87	0.74
L4	3.69	0.72	3.68	0.64	3.40	0.64	3.04	0.64	3.45	0.70
L3	4.58	0.97	4.33	0.83	4.12	1.02	3.54	0.84	4.15	0.99
L2	2.80	0.99	3.47	0.76	2.79	0.75	2.81	0.73	2.97	0.85
L1	2.99	0.88	3.16	0.64	2.76	0.64	2.93	0.77	2.96	0.74

Reprinted with permission from Eltink et al. [17, 27]

For clinical crown height, independent *t*-tests found teeth with recession were significantly larger than the clinical crown height measurement for the non-recession teeth (Table 3).

**Table 3:** Independent *t*-tests comparing the no recession to recession subgroups using the clinical crown height measure for the combined four groups

Tooth class	Group	*n*	Mean	SD	*t*	*p*
U6	No recession	96	6.41	0.85	− 10.41	0.000*
	Recession	30	8.25	0.83		
U5	No recession	105	6.94	0.86	− 7.57	0.000*
	Recession	31	8.28	0.88		
U4	No recession	96	7.94	0.82	− 6.62	0.000*
	Recession	33	9.06	0.91		
U3	No recession	102	9.73	0.89	− 7.36	0.000*
	Recession	42	11.02	1.10		
U2	No recession	103	8.66	0.99	− 5.27	0.000*
	Recession	23	9.80	0.63		
U1	No recession	107	10.13	1.00	− 5.31	0.000*
	Recession	19	11.49	1.17		
L6	No recession	93	6.56	0.71	− 5.88	0.000*
	Recession	13	7.92	1.19		
L5	No recession	101	7.24	0.82	− 6.07	0.000*
	Recession	23	8.41	0.92		
L4	No recession	96	8.14	0.75	− 6.35	0.000*
	Recession	23	9.25	0.75		
L3	No recession	104	9.50	1.16	− 7.08	0.000*
	Recession	30	11.11	0.80		
L2	No recession	114	8.22	0.92	− 6.05	0.000*
	Recession	11	9.98	0.97		
L1	No recession	105	7.99	0.98	− 7.17	0.000*
	Recession	22	8.58	0.74		

*p < .05, indicating a difference and comparing the recession and no recession subgroups—a true positive value

Reprinted with permission from Eltink et al. [17, 27]

For gingival margin-papillae measure, teeth in the recession subset were significantly larger than non-recession for all tooth classes except for the lower lateral incisor (Table 4).

**Table 4:** Independent *t*-tests comparing the no recession to recession subgroups using the gingival margin-papillae measure for the combined four groups

Tooth class	Group	*n*	Mean	SD	*t*	*p*
U6	No recession	96	2.33	0.73	− 7.53	0.000*
	Recession	30	3.51	0.81		
U5	No recession	105	2.45	0.70	− 5.76	0.000*
	Recession	31	3.36	0.88		
U4	No recession	96	3.00	0.69	− 6.74	0.000*
	Recession	33	3.95	0.79		
U3	No recession	102	4.08	0.79	− 6.23	0.000*
	Recession	42	5.22	1.28		
U2	No recession	103	3.28	1.05	− 2.62	0.000*
	Recession	23	3.89	0.61		
U1	No recession	107	3.84	1.05	− 3.40	0.000*
	Recession	19	4.94	2.17		
L6	No recession	93	2.51	0.61	− 6.51	0.000*
	Recession	13	3.89	1.27		
L5	No recession	101	2.74	0.63	− 5.13	0.000*
	Recession	23	3.57	0.94		
L4	No recession	96	3.39	0.69	− 3.15	0.000*
	Recession	23	3.91	0.84		
L3	No recession	104	3.91	0.91	− 5.71	0.000*
	Recession	30	5.03	1.01		
L2	No recession	114	2.93	0.82	− 1.18	0.239
	Recession	11	3.27	1.33		
L1	No recession	105	2.83	0.66	− 5.25	0.000*
	Recession	22	3.76	1.05		

*p < .05, indicating a difference and comparing the no recession and recession subgroups—a true positive value

Reprinted with permission from Eltink et al. [17, 27]

The overall data set (*N* = 1467 teeth) was then separated into two subsets on the basis of the tooth wear index for each tooth. The first subset, the normal wear teeth (*N* = 1230 teeth), was comprised of all teeth that were scored with a value of 2 or less on Hooper’s tooth wear index since moderate tooth wear may be considered normal [16]. The second subset, called the “severe wear teeth” (*N* = 237 teeth), comprised of all teeth that were scored with a value of 3 or greater on the tooth wear index (Fig. 2).

The results of the independent *t*-tests for clinical recession between the normal wear and severe wear subsets revealed no significant differences for any tooth class (Table 5).

**Table 5:** Independent *t*-tests comparing the measure of clinical recession in the normal wear and severe wear subsets

Tooth class	Group	*n*	Mean	SD	*t*	*p*
U6	Normal wear	107	0.29	0.59	0.12	0.906
	Severe wear	12	0.27	0.47		
U5	Normal wear	110	0.22	0.41	1.88	0.062
	Severe wear	16	0.03	0.12		
U4	Normal wear	109	0.27	0.48	0.86	0.393
	Severe wear	9	0.13	0.35		
U3	Normal wear	110	0.32	0.54	0.55	0.581
	Severe wear	18	0.25	0.43		
U2	Normal wear	106	0.16	0.36	0.65	0.515
	Severe wear	11	0.09	0.30		
U1	Normal wear	97	0.18	0.43	1.92	0.058
	Severe wear	22	0.00	0.00		
L6	Normal wear	78	0.11	0.42	− 0.82	0.415
	Severe wear	44	0.18	0.48		
L5	Normal wear	104	0.18	0.40	0.59	0.557
	Severe wear	17	0.12	0.33		
L4	Normal wear	107	0.21	0.44	− 0.63	0.532
	Severe wear	10	0.31	0.70		
L3	Normal wear	103	0.26	0.51	1.48	0.141
	Severe wear	29	0.12	0.28		
L2	Normal wear	102	0.09	0.30	0.78	0.439
	Severe wear	23	0.04	0.20		
L1	Normal wear	97	0.19	0.41	1.14	0.256
	Severe wear	26	0.09	0.28		

*p* < .05, comparing the normal wear and severe wear subgroups; no significant difference was found in the clinical recession due to tooth wear

Reprinted with permission from Eltink et al. [17, 27]

For clinical crown height, significant differences were found between the normal wear and the severe wear teeth for all tooth classes except the maxillary first molar. For all other tooth classes, the clinical crown height measurements for teeth in the normal wear subset were larger than the measurements for teeth in the severe wear subset (Table 6). However, for gingival margin-papillae, no significant differences were found between the normal wear teeth and the severe wear teeth (Table 7).

**Table 6:** Independent *t*-tests comparing the clinical crown height for the normal wear and severe wear subsets measured by the clinical crown height for the combined four groups

Tooth class	Group	*n*	Mean	SD	*t*	*p*
U6	Normal wear	107	6.75	1.09	0.10	0.923
	Severe wear	12	6.71	1.25		
U5	Normal wear	110	7.21	1.00	2.29	0.023*
	Severe wear	16	6.62	0.69		
U4	Normal wear	109	8.20	0.94	3.21	0.002*
	Severe wear	9	7.18	0.52		
U3	Normal wear	110	10.11	1.13	3.68	0.000*
	Severe wear	18	9.09	0.84		
U2	Normal wear	106	8.89	1.00	3.16	0.002*
	Severe wear	11	7.88	1.13		
U1	Normal wear	97	10.41	1.12	3.13	0.002*
	Severe wear	22	9.63	0.72		
L6	Normal wear	78	6.78	0.92	2.31	0.023*
	Severe wear	44	6.40	0.77		
L5	Normal wear	104	7.50	0.84	3.56	0.001*
	Severe wear	17	6.66	1.25		
L4	Normal wear	107	8.37	0.83	3.52	0.001*
	Severe wear	10	7.40	0.87		
L3	Normal wear	103	10.00	1.28	4.14	0.000*
	Severe wear	29	8.93	0.99		
L2	Normal wear	102	8.42	1.02	3.04	0.003*
	Severe wear	23	7.72	0.86		
L1	Normal wear	97	8.41	1.01	4.65	0.000*
	Severe wear	26	7.37	1.04		

*p < .05, comparing the clinical crown between the normal wear and severe wear subgroups and showing the significant differences for all tooth classes due to a decrease in the clinical crown height in the severe wear group—a false positive value

Reprinted with permission from Eltink et al. [17, 27]

**Table 7:** Independent *t*-tests comparing the gingival margin-papillae measurements for the normal wear and severe wear subsets for the combined four groups

Tooth class	Group	*n*	Mean	SD	*t*	*p*
U6	Normal wear	107	2.51	0.82	− 1.33	0.187
	Severe wear	12	2.87	1.04		
U5	Normal wear	110	2.62	0.82	1.07	0.285
	Severe wear	16	2.40	0.53		
U4	Normal wear	109	3.22	0.82	1.51	0.134
	Severe wear	9	2.78	0.47		
U3	Normal wear	110	4.39	1.08	0.46	0.649
	Severe wear	18	4.27	0.99		
U2	Normal wear	106	3.39	1.01	0.96	0.341
	Severe wear	11	3.08	1.19		
U1	Normal wear	97	3.90	1.14	0.17	0.868
	Severe wear	22	3.86	0.81		
L6	Normal wear	78	2.62	0.88	0.06	0.950
	Severe wear	44	2.61	0.76		
L5	Normal wear	104	2.90	0.74	0.31	0.761
	Severe wear	17	2.93	0.98		
L4	Normal wear	107	3.45	0.71	− 0.10	0.920
	Severe wear	10	3.48	0.86		
L3	Normal wear	103	4.20	1.01	1.59	0.114
	Severe wear	29	3.87	0.91		
L2	Normal wear	102	2.98	0.88	0.19	0.851
	Severe wear	23	2.94	0.87		
L1	Normal wear	97	2.99	0.74	1.32	0.189
	Severe wear	26	2.77	0.78		

*p* < .05, indicating that the gingival margin-papillae measure comparing the normal wear and severe wear subgroups was not different because of tooth wear—a true negative value

Reprinted with permission from Eltink et al. [17, 27]

Table 8 lists the frequencies of teeth with and without clinical recession and advanced tooth wear in the four demographic groups.

**Table 8:** Clinical recession and tooth wear frequencies by the demographic group

Demographic group	Total teeth	Number and (%) of teeth with clinical recession	Number of teeth with severe tooth wear (%)
Caucasian male	684	134 (20)	126 (18)
Caucasian female	665	133 (20)	117 (18)
African-American male	668	130 (19)	65 (10)
African-American female	662	41 (6)	56 (8)

Reprinted with permission from Eltink et al. [17, 27]

## Discussion

The means and standard deviations for the clinical crown height and gingival margin-papillae measurements (Tables 1 and 2) are reported to allow for comparison to other samples in the future. While clinical crown height has been studied extensively in children and adolescents [11, 12], the gingival margin-papillae measurement has never been evaluated. With values for adults reported by race and by gender, by individual tooth class from the central incisor to the first molar, the effects of other variables on the architecture of the gingiva can be studied. All measurements were done by one investigator, 10 days apart, and showed a significant correlation at the 0.01 level. However, multiple measures done by a series of investigators would be necessary to determine the reproducibility of the measures.

The focus of this study was to determine if either clinical crown height or gingival margin-papillae could serve as a quantitative indirect measure to detect gingival recession. The clinical crown height measurement detected differences in the position of the gingival margins between the recession and non-recession teeth for all tooth classes, therefore giving a *true positive* result where a difference was detected and a difference did exist (Table 3). The gingival margin-papillae measurement revealed similar results, detecting a difference between the recession and non-recession teeth, another true positive finding (Table 4).

The ability of a measurement to detect a true positive is useless unless the same measurement can provide a *true negative* result, finding no difference where no difference exists. To test this principle, the overall data set was divided into two subsets—teeth with normal wear and teeth with severe wear. There were no differences in measures of clinical recession with respect to tooth wear (Table 5). The clinical crown height measurement (Table 6) found differences between the subsets with the values for the normal wear teeth being larger than the values for the severe wear teeth, which is a *false positive* result since there was no difference in the gingival architecture. However, in short-term studies, for example the effect of orthodontic treatment, attrition is not likely to be important.

**Fig. 3:**
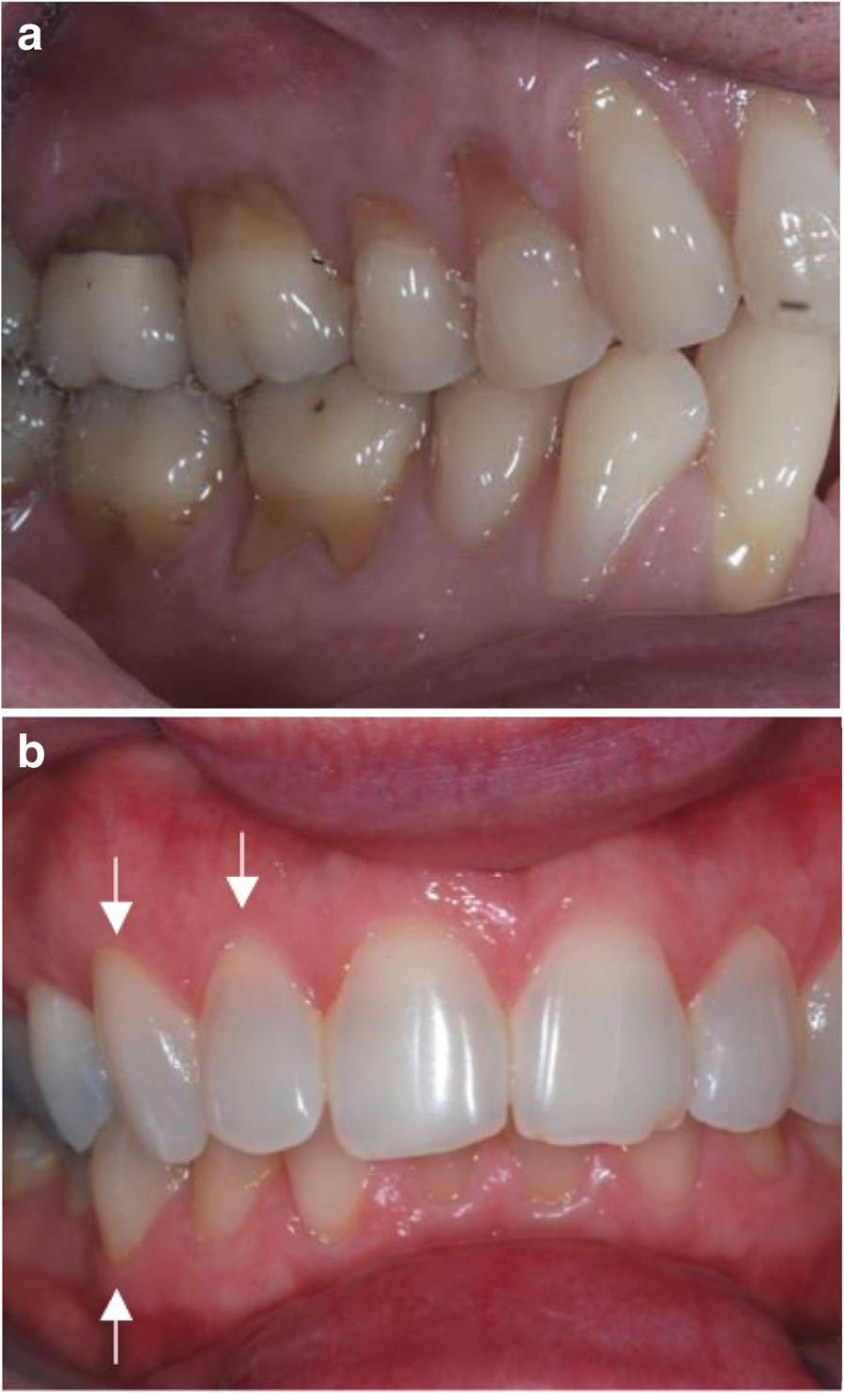
**a**, **b** Patient with advanced gingival recession. In patients who are otherwise periodontally healthy the papillae maintains its normal position while the free gingival margin recedes, exposing the root cementum. **b** Patient with prodromal recession: Teeth with arrows don't how cemental exposure but show an increase in the gingival margin papillae measurement

The gingival margin-papillae measurement, however, found no difference where no difference in the position of the gingival margin existed, a true negative finding (Table 7). The use of the adjacent papillae as landmarks for the measurement of the position of the gingival margin eliminates the problems that result from tooth wear. It is important to note that despite advanced recession, when the periodontium is otherwise healthy, the interproximal papillae maintain their position while the free gingival margin recedes (Fig. 3a).

The gingival margin-papillae measurement may prove most useful in diagnosing *prodromal recession*, when the gingiva recedes but the root cementum has not been exposed (Fig. 3b). Many clinicians intuitively visualize this measure when they diagnose a *tendency* for gingival recession.

When comparing the clinical crown height to the gingival margin-papillae measurement, the latter not only has the advantage of avoiding the problems attendant with attrition but also sidesteps the variability of tooth size in individuals due to race, sex, and genetic expression.

Bruxism is the main cause of tooth wear and is associated with excessive occlusal force that some clinicians feel will cause abfraction and can lead to gingival recession [18]. The data in this paper indicates that attrition was not associated with an increase in both clinical recession and the gingival margin-papillae measures (Tables 5 and 7). Therefore, any association of bruxism or abfraction to gingival recession was not demonstrated in this study.

The Miller index, used by periodontists to classify gingival recession, has four grades [19]. Grades 3 and 4 involve interproximal bone loss so that only grades 1 and 2 are appropriate measures in subjects otherwise free of periodontal disease. Note that the Miller index is not a quantitative measure of gingival recession but it is useful in describing the severity of recession into four clinical categories. The difference between Miller grades 1 and 2 is the amount of attached gingiva. In this paper, we did not attempt to measure this level. Wennström has presented evidence that the amount of attached gingiva is not associated with the development of gingival recession and that minimal attached gingiva is sufficient to prevent recession [20].

Gingival recession is common in the adult population with an otherwise healthy periodontium and was observed in 20% of the teeth in the present study. What is the cause of this variability of expression of naturally occurring recession? Undoubtedly, one factor is over-zealous tooth brushing [21]. The genetically determined thickness of the alveolar bone and soft tissue—the biotype—may be an important variable of naturally occurring gingival recession [22], especially if associated with bone dehiscences [23]. However, Cook et al. found that periodontal biotype was unrelated to labial gingival recession [24]. Frenal pull [25] and plaque and calculus [26] are local factors that can result in gingival recession. It would appear that age-related gingival recession seen in many adults is multifactorial and any of the above factors may be contributory.

How useful will the measures of gingival recession and tooth wear be in identifying differences in incidence when the study group is divided by gender and race? Table 8 lists the percentage of teeth with clinical recession and severe tooth wear for the four demographic groups. African-American females have less clinical recession than African-American males (6 vs 19%) as well as in Caucasian males and females (20%). African-American males and females have one half as much tooth wear as their Caucasian counterparts. A detailed statistical analysis confirming these differences appears in previous publications [17, 27].

Volchansky and Cleaton-Jones [11] studied clinical crown height in 18-year-old Caucasians, and Bassey [28] studied Nigerians aged 29.7 years. These authors, however, were not focused on the problem of gingival recession.

One of the limitations of this paper is the grouping of all patients between the age of 20 and 59 years. A larger sample would be required to demonstrate the level of recession seen in each age group, which has been shown to increase with age [1].

Applying the gingival margin-papillae measure intraorally would be difficult, and therefore, study models are required. However, the use of intraoral scans could make accurate measurements possible without impressions and study models, as the scans are easily converted into digital models [29].

The three measures studied in this paper have strengths and weaknesses. Gingival recession measured by a periodontal probe to the nearest millimeter is clinically useful but is semi-quantitative, cannot measure the earliest stages of recession prior to cemental exposure, and is dependent upon visualization of the cemento-enamel junction (CEJ) which at times can be difficult to locate [30]. Crown length is fine for short-term studies but, as shown in this paper, will give false readings due to tooth wear with long-term studies. The gingival margin-papillae measurement is more time consuming to use and is not appropriate once interproximal bone loss from periodontal disease is present, but it is not dependent upon visualizing the CEJ and not affected by attrition. When orthodontic study models, or virtual models are available, both crown length and the gingival margin-papillae can be quantified to at least a tenth of a millimeter. Gingival hyperplasia is often seen at the time of debonding but usually recedes by the time records are taken. This hyperplasia will affect all three measures, but papillary hyperplasia will be more of a problem for the gingival papilla measure.

The quantitative measures presented in this paper will be valuable in the study of gingival recession that may be associated with orthodontic tooth movement that challenges the limits of the alveolus, for example lower incisor proclination [31–34] and buccal expansion [34, 35]. The gingival margin-papillae measure would be useful in following surgical procedures to correct gingival recession [36] especially followed over time.

Recent interest in expansion of the anterior and posterior dental alveolus has been stimulated by the Damon self-ligating bracket system [37, 38] and the use of Invisalign for expansion [39]. The standards presented in this paper would be useful in evaluating recession in the above treatments, especially long-term results.

Accelerated osteogenic orthodontic techniques involve flapping of the gingiva, corticotomy, and usually bone grafting in an effort to increase the width of the alveolus for expansion [40]. We need more data, both short- and long-term, to evaluate the potential impact on gingival recession.

The power of measures of gingival recession and tooth attrition is demonstrated in the differences seen in the four demographic groups studied (Table 8). This will be very useful in epidemiological studies, for example comparison of subjects with abrasive versus soft diets.

Clinical recession, clinical crown height, and gingival margin-papillae measurements all have advantages and disadvantages and, therefore, could be considered together to provide an accurate and precise assessment of the architecture of the gingival tissue.

## Conclusions

1. Two quantitative measures that indirectly measure gingival recession—clinical crown height and the gingival margin-papillae measure—are presented for four demographic groups (Caucasian and African-American males and females) for all teeth from the central incisors to and including the first molars.
2. Segregating the total group by teeth with recession and without recession demonstrates that both measures report an increased value associated with recession, a positive finding.
3. Segregating the total group by teeth with severe wear and normal wear demonstrates a decrease in the clinical crown height measure due to the shorter tooth which could mask gingival recession. The gingival margin-papillae measure, however, was not altered by tooth wear.
4. Tooth wear usually caused by bruxism was not associated with an increase in gingival recession.
5. Measures for gingival recession and tooth wear were different for the four demographic groups studied, indicating the power of these measures in future epidemiological studies.
6. The clinical crown height and gingival margin-papillae measures can be used to precisely quantify gingival recession following orthodontic and/or periodontal procedures.

## References

[1] Serino G, Wennström J, Lindhe J, Eneroth L (1994). The prevalence and distribution of gingival recession in subjects with high standards of oral hygiene. J Clin Periodontal.

[2] Schroeder HE, Listgarten MA (1997). The gingival tissues: the architecture of periodontal protection. Periodontol.

[3] Listgarten MA (1972). Normal development, structure, physiology and repair of gingival epithelium. Oral Sci Rev.

[4] LaVacca MI, Tarnow DP, Cisneros GJ (2005). Interdental papilla length and the perception of aesthetics. Pract Proced Aesthet Dent.

[5] Kokich VG (1996). Esthetics: the orthodontic–periodontic connection. Semin Orthod.

[6] Tarnow DP, Magner AW, Fletcher P (1992). The effect of the distance from the contact point to the crest of bone on the presence or absence of the interdental papilla. J Periodontol.

[7] Löe H, Goldman HM, Cohen HW (1968). Periodontal therapy.

[8] Newman MG, Takei H, Klokkeveld PR, Carranza FA, editors. Carranza’s clinical periodontology. 12th ed. St. Louis: Elsevier; 2014.

[9] Hara K, Hosada H (1976). Gingival architectural forms in periodontal diseases. Bull Tokyo Med Dent Univ.

[10] Watson PJ (1984). Gingival recession. J Dent.

[11] Volchansky A, Cleaton-Jones P (2001). Clinical crown height (length)—a review of published measurements. J Clin Periodontol.

[12] Powell RN, McEniery TM (1981). Disparities in gingival height in the mandibular central incisor region of children aged 6-12 years. Community Dent Oral Epidemiol.

[13] Gorman WJ (1967). Prevalence and etiology of gingival recession. J Periodontol.

[14] Albandar JM, Kingman A (1999). Gingival recession, gingival bleeding, and dental calculus in adults 30 years of age and older in the United States, 1988-1994. J Periodontol.

[15] Löe H, Anerud A, Boysen H (1992). The natural history of periodontal disease in man: prevalence, severity, extent of gingival recession. J Periodontol.

[16] Hooper SM, Meredith N, Jagger DC (2004). The developments of a new index for measurement of incisal/occlusal tooth wear. J Oral Rehabil..

[17] Eltink AP. Gingival architecture of adult orthodontic patients: a new method of assessment of gingival recession. University of Illinois at Chicago, Pro Quest, UMI Dissertation Publication. 2005;1425218.

[18] Lyons K (2001). Aetiology of abfraction lesions. N Z Dent J.

[19] Pini-Prato G (2011). The Miller classification of gingival recession; limits and drawbacks. J Clin Periodontal..

[20] Wennström JL (1987). Lack of association between width of attached gingiva and the development of gingival recessions: a 5-year longitudinal study. J Clin Periodont..

[21] Checchi L, Daprile G, Gatto MR (1999). Gingival recession and tooth brushing in an Italian school of dentistry: a pilot study. J Clin Periodont..

[22] Fu JH, Yeh CY, Chan HL (2010). Tissue biotype and its relation to the underlying bone morphology. J Periodontol..

[23] Löst C (1984). Depth of alveolar bone dehiscences in relation to gingival recessions. J Clin Periodont..

[24] Cook DR, Mealey RL, Verrett RG, Mills MP, Noujeim ME, Lasho DL, Cronim RJ (2011). Relationship between clinical periodontal biotype and labial plate thickness: an in vitro study. Int J Perio & Restorative. Dent..

[25] Trott JR, Love B (1966). An analysis of local recession in 766 Winnipeg high school students. Dent Practice..

[26] van Palenstein Helderman WH, Lembariti BS, van der Weijden GA (1998). Gingival recession and its association with calculus in subjects deprived of prophylactic dental care. J Clin Periodont..

[27] Eltink AP, Handelman CS, BeGole EA. A new measure of gingival recession and the significance of attriction, gender, and race. In, McNamara JA Jr, Hatch N, Kapila SD (eds): Effective and efficient orthodontic tooth movement. Craniofacial Growth Series, Volume 48. Ann Arbor, Center for Human Growth and Development, University of Michigan; 2011. pp. 353-75.

[28] Bassey IEE (1991). Clinical crown heights of permanent teeth in Nigerians. Afr Dent J.

[29] Avagon MLC, Pontes LF, Bicharah LM, Flores-Mir C, Narmando D (2016). Validity and reliability of intraoral scanners compared to conventional gypsum models measurements: a systemic review. Euro J Ortho.

[30] Smith RG (1997). Gingival recession. Reappraisal of the enigmatic condition and a new index for monitoring. J Clin Periodont.

[31] Allais D, Melsen B (2003). Does labial movement of lower incisors influence the level of the gingival margin? A case-control study of adult orthodontic patients. Eur J Orthod.

[32] Ruf S, Hansen K, Pancherz H (1995). Does orthodontic proclination of lower incisors in children and adolescents cause gingival recession. Am J Orthod Dentofac Orthop.

[33] Renkema AM, Fudalej PS, Renkema A, Bronkhorst E, Katsaros C (2012). Gingival recession and the change in inclination of mandibular incisors during orthodontic treatment. Eur J Orthod.

[34] Morris JW, Campbell PM, Tadlock (2017). Prevelance of gingival recession after orthodontic tooth movements. Am J Orthod Dentofac Orthop.

[35] Handelman CS, Wang L, Begole EA, Haas A (2000). Nonsurgical rapid maxillary expansion in adults: report on 47 cases using the Haas expander. Angle Orthod.

[36] Oates TW, Robinson M, Gunsolley (2003). Surgical therapies for the treatment of gingival recession. A systematic review. Ann Periodontol.

[37] Bascifici FA, Akin M, Iberi Z, Bayram S (2014). Long-term stability of dentoalveolar, skeletal, and soft tissue changes after non-extraction treatment with a self-ligating system. Korean J Orthod.

[38] Atik E, Semra C (2014). An assessment of conventional and self-ligating brackets in class I maxillary constriction patients. Angle Orthod.

[39] Houle J-P, Piedade L, Todescan R, Pinheiro HSL (2017). The predictability of transverse changes with Invisalign. Angle Orthod.

[40] Wilco MT, Wilco WM, Pulver JJ, Bissada NF, Bouquot JE (2009). Accelerated osteogenic orthodontics techniques: a 1-stage surgically facilitated rapid orthodontic technique with alveolar augmentation. J Oral Maxillofac Surg.

